# Application of Machine Learning for Calibrating Gas Sensors for Methane Emissions Monitoring

**DOI:** 10.3390/s23249898

**Published:** 2023-12-18

**Authors:** Ballard Andrews, Aditi Chakrabarti, Mathieu Dauphin, Andrew Speck

**Affiliations:** Schlumberger-Doll Research, Cambridge, MA 02139, USA; achakrabarti2@slb.com (A.C.); mdauphin@slb.com (M.D.); aspeck@slb.com (A.S.)

**Keywords:** methane emissions, machine learning, Gaussian process regression, artificial neural nets, MO_X_ sensors

## Abstract

Methane leaks are a significant component of greenhouse gas emissions and a global problem for the oil and gas industry. Emissions occur from a wide variety of sites with no discernable patterns, requiring methodologies to frequently monitor these releases throughout the entire production chain. To cost-effectively monitor widely dispersed well pads, we developed a methane point instrument to be deployed at facilities and connected to a cloud-based interpretation platform that provides real-time continuous monitoring in all weather conditions. The methane sensor is calibrated with machine learning methods of Gaussian process regression and the results are compared with artificial neural networks. A machine learning approach incorporates environmental effects into the sensor response and achieves the accuracies required for methane emissions monitoring with a small number of parameters. The sensors achieve an accuracy of 1 part per million methane (ppm) and can detect leaks at rates of less than 0.6 kg/h.

## 1. Introduction

Most methane emissions in the oil and gas (O&G) sector occur during the production, transmission, and storage of oil and gas. Methane contributes around 50% of the total carbon dioxide equivalent (CO_2_e) emitted by oil and gas production with the remainder from CO_2_. However, due to methane’s high greenhouse warming potential, the volume of methane emitted is almost 80 times lower (in a 20-year timeframe) compared to CO_2_ and is thus the simplest to abate [1,2]. The O&G industry is responsible for approximately one-fifth of the total methane emissions [3,4]. Many O&G companies have seized the initiative to measure and quantify their emissions, both to meet the proposed US regulations and to meet their own carbon reduction goals. A major portion of the methane emissions from upstream and downstream operations arises from a small number of large emission events. An emission event is a discrete period when methane is released into the atmosphere from a specific piece of equipment within a finite time window. These emissions originate from a variety of sites, and recent data suggest that many emission events are intermittent [5,6]. Currently, there is no way to predict which facilities are likely to release large quantities of methane and thus all sites along the upstream production network must be monitored. Continuous monitoring using methane emissions detectors installed permanently at a site offers an effective way to identify, quantify, and repair intermittent emissions. However, installing high-performance emissions detectors across many diverse sites can be economically challenging.

**Figure 1:**
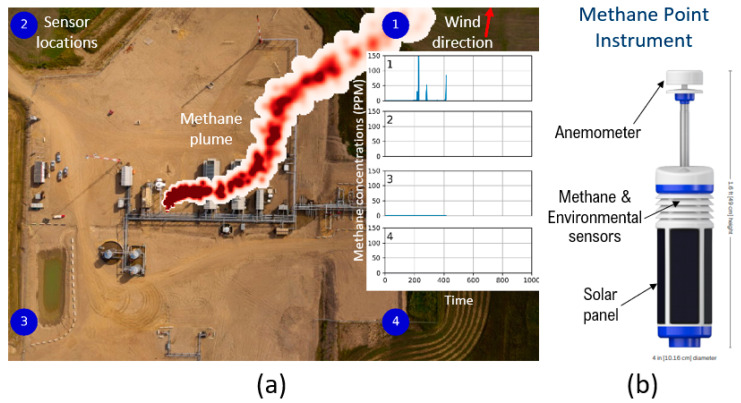
(**a**) Typical system layout showing four methane point instruments (1–4), along the boundary of an O&G facility. Each sensor continuously reports methane concentrations and meteorological data to a cloud gateway after which algorithms running in the cloud interpret these time-series concentrations and meteorological data to determine if a leak is occurring and, if so, establish its anticipated leak rate and location. The inset shows the concentration measured at each sensor location. (**b**) The methane point instrument consists of the methane sensor (MOx), temperature, and humidity sensors, mounted inside a housing with filters to prevent accumulation of dust, water, and snow; an anemometer atop the pole, a wrap-around solar panel; and the battery and electronics inside its main body.

SLB has developed a cost-optimized, continuous methane emissions detector connected through the Internet of Things (IoT), capable of being rapidly deployed, which enables continuous monitoring with a low incidence of false positives [7,8]. The core of the system is a network of battery-powered methane sensors deployed at fixed locations around the facility (Figure 1), which meets the requirements of accuracy, limit of detection, power consumption, form factor, and cost. Measurements of methane concentration and meteorological conditions are continuously recorded. The data are sent for interpretation to a cloud platform via a secure gateway where they are inverted based on a plume dispersion model to confirm the leak position and emission rate. The plume model provides the link between the methane concentration measured via the methane sensors and leak location, duration, and rate [7,8,9]. The performance of the system has been validated and verified with a lengthy and rigorous testing procedure at a facility that can generate controlled methane releases. This paper describes the development of the fit-for-purpose sensor in more detail and thus complements our previous publications [7,8,9].

The methane sensors’ performance is key to the system as it determines the lowest leak rate the system can detect, and its localization and quantification accuracy. Beyond the obvious requirements on accuracy, limit of detection, and reproducibility, there are several other requirements including power consumption, form factor, and cost. Based on our investigations of over forty available sensor candidates, we selected semiconducting metal oxide (MOx) sensors for our detection system. The primary factors influencing our selection of MOx sensors were commercial availability, low power consumption, and economic viability for upscaling to large quantities. Research on the increased sensitivity of MOx sensors may allow for enhanced performance [10].

## 2. Materials and Methods

### 2.1. MOx Sensor Calibration

**Figure 2:**
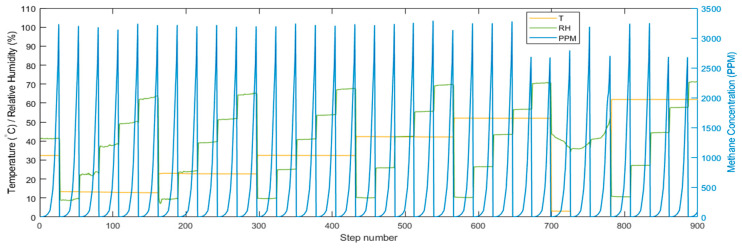
During a calibration run, the methane concentration (in parts per million, ppm) is ramped for each relative humidity (RH) and temperature (T) combination. The right axis shows the readings of methane from the optical reference sensor.

The MOx sensor response for a given methane concentration depends on the temperature (T) and relative humidity (RH). Sensor calibration is performed in an environmental chamber (Test Equity 101H, Moorpark, CA, USA) where T and RH are accurately controlled, and methane concentration is varied using LabVIEW (National Instruments, Austin, TX, USA) and independently determined via a sub-ppm optical reference sensor. The environmental chamber has a temperature range of −30 °C to 130 °C, and a humidity range of 10% to 95% relative humidity. The optical reference sensor uses mid-infrared absorption spectroscopy with a laser diode. It has a range of 10 ppb–10,000 ppm with a precision of 2 ppb/s. The active element in the MOx sensor is heated to 200–500 °C to increase the reduction–oxidation reaction rate with adsorbed gases and reduce sensitivity to moisture. A mixture of 5% methane gas in nitrogen is mixed with air from a zero-air generator to produce contamination-free gas and obtain desired concentrations of methane in the environmental chamber using precise mass flow controllers (MFC, Alicat Scientific, Tucson, AZ, USA). We have zero air regulated by a single MFC and methane is regulated using 2 MFCs, one for the coarse flow rates and one for the finer flow rates, which helps us control methane concentrations in the sub-ppm range. The measurement range is 0.0002 to 50 SLPM and the flow measurement accuracy is ±0.6% of reading. In a calibration run, at each step, temperature and humidity are fixed (i.e., fixed absolute humidity) while the methane concentration (ppm) is increased (Figure 2). 

The response of an MOx sensing element exposed to a gas can be described by an empirical power law (Equation (1)):

(1) $$R\;=\;{\;R}_{0}\;{\left(1\;+\;C_{\mathrm{gas}}K_{\mathrm{gas}}\right)}^{-\beta }$$

where R_0_ is the resistance in the absence of target gas, K_gas_ is the sensitivity to a particular analyte, β is the coefficient of response, and C_gas_ is the gas concentration. K_gas_ and β depend on the analyte concentration and the sensing material. Environmental conditions (temperature and humidity) also significantly impact the response; this dependence is not explicit in Equation (1) but must be considered.

**Figure 3:**
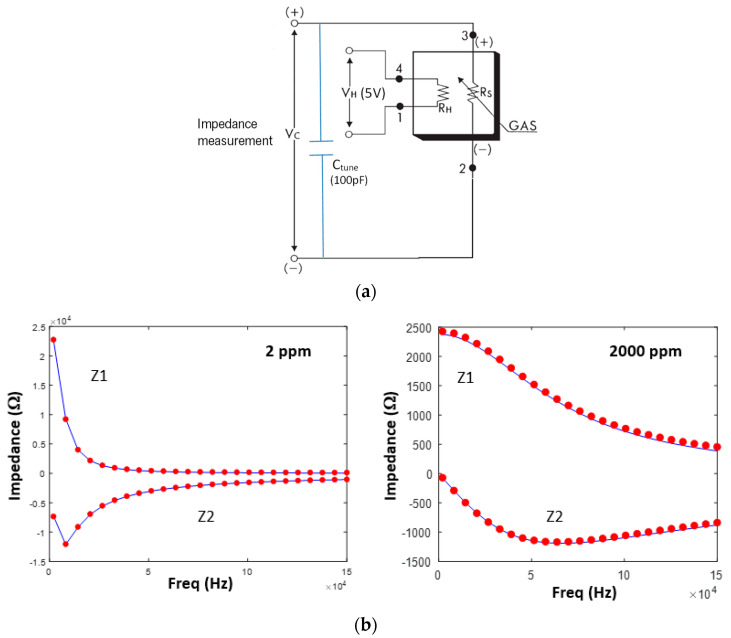
(**a**) MOx resonant resistance circuit. Impedance analyzer is connected across V_C_ (+) and V_C_ (−); (**b**) Real (Z1) and imaginary (Z2) parts of the impedance for two different methane concentrations. Red dots are the measured frequencies and blue lines are curve fits (Equation (2)).

**Figure 4:**
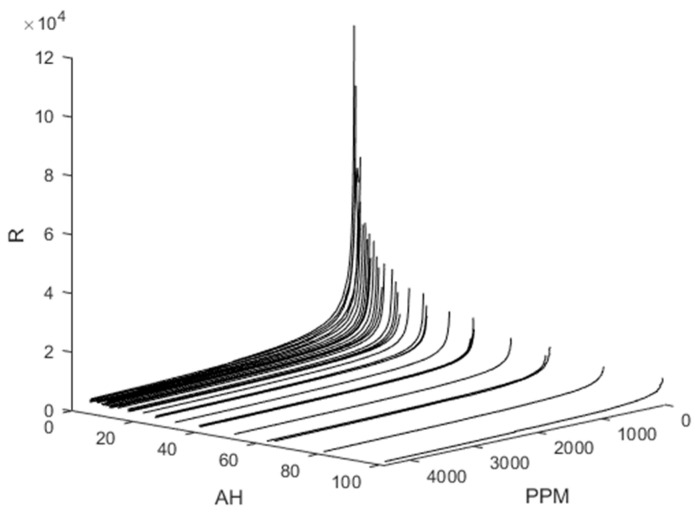
3D plot of sensor resistivity (R) vs. absolute humidity (AH) and measured methane concentration (ppm) of reference methane gas analyzer.

The resonant AC circuit shown in Figure 3a extends the dynamic range, where R_S_ is the sensor resistance, V_C_ is the voltage across the sensor and tuning capacitor, R_H_ is the heater resistance, and V_H_ is the heater voltage. When the real (Z_1_) and imaginary (Z_2_) parts of the impedance (Equation (2) and Figure 3b) are solved simultaneously for R, a unique curve for each temperature and relative humidity is obtained (Figure 4). The set of curves can be fit with Equation (1) provided R_0_, K_gas_, and β are not held fixed. Additional parametrization is required to account for environmental conditions [11,12,13].

(2) $$Z1\;=\;R/\left[1\;+\;{\left(2\pi fCR\right)}^{2}\right]\;\;\;\;\;\;\;\;\;Z2\;=\;-R^{2}C2\pi f/\left[1\;+\;{\left(2\pi fCR\right)}^{2}\right]\;$$

Instead, the approach adopted here is to model this dependence using machine learning (ML). ML has been explored for MOx gas sensing [14,15], but to our knowledge, has not been commercially adopted specifically for methane emissions monitoring. An advantage of using an ML approach is that T and RH (or alternatively AH), are paired with R as predictors of the methane concentration in parts per million (ppm). In this study, we examine two types of ML regression methods: Gaussian process regression (GPR) and artificial neural networks (ANN). Our primary focus in the paper is on Gaussian process regression; ANNs are included chiefly for comparison. 

### 2.2. Gaussian Process Regression

GPR is a nonlinear, nonparametric regression technique defined by a covariance function or kernel ***k***(***x***, ***x***’). GPR models predict the values of a function ***y***(***x***), together with a mean and uncertainty, at arbitrary locations from the observations, ***y***(***x***_***n***_). The function ***y***(***x***) is approximated as a linear combination of basis functions $k\left(x,\;x_{m}\right)$ at arbitrary locations $\tilde{y}(x)$; the matrix form is given by Equation (3), where ***c_m_*** are the weights:

(3) $$\tilde{y}\left(x\right)\;=\;\sum_{m\;=\;1}^{M}c_{m}k\left(x,\;x_{m}\right)\;=\;c^{T}k\left(x,\;x_{m}\right)$$

Equation (4) is one of the most widely used covariance functions, the squared exponential, also referred to as a radial basis function. The hyperparameters in the kernel are the standard deviation σ_f_ and characteristic length scale σ_l_, which governs how quickly correlations between the data points decay with distance. The initial values of σ_l_ and σ_f_ can be determined from the data. For example, the mean and standard deviation of R and T and RH are used for σ_l_, and the standard deviation of the response (ppm) for σ_f_/√2.

(4) $$k\left(x_{i},\;x_{j}|\theta \right)\;=\;\sigma_{f}^{2}exp\left[-\frac{1}{2}\frac{{\left(x_{i}\;-\;x_{j}\right)}^{T}\left(x_{i}\;-\;x_{j}\right)}{\sigma_{l}^{2}}\right]$$

Non-isotropic kernels, also referred to as automatic relevance determination (ARD) kernels, have separate length scales for each of the predictors. Equation (5) is the non-isotropic form of the squared exponential kernel in Equation (4):

(5) $$k\left(x_{i},\;x_{j}|\theta \right)=\sigma_{f}^{2}exp\left[-\frac{1}{2}\sum_{m\;=\;1}^{d}\frac{{\left(x_{im}\;-\;x_{jm}\right)}^{2}}{\sigma_{m}^{2}}\right]$$

Testing various kernels will determine which kernel or combination thereof yields optimal performance. Regularization is controlled by the parameter σ_f_ which imposes smoothness.

We give a brief outline of the log marginal likelihood (LML) method for the isotropic case. More details can be found in reference [16]. In the LML approach, the coefficients ***c_m_*** are found by minimizing a loss function ℒ with a regularization term, $c^{T}K_{MM}c$ where $\left[K_{NM}{]}_{nm}\;=\;k(x_{n},\;x_{m}\right)$ is the matrix of covariance functions. In matrix form, the loss function is written as:

(6) $$ℒ\;=\;{\left(y\;-\;K_{NM}c\right)}^{T}\sum^{-1}\left(y\;-\;K_{NM}c\right)\;+\;c^{T}K_{MM}c$$

when Equation (6) is differentiated and solved for **c** we obtain:

(7) $$c\;=\;{\left(K_{MM}\;+\;K_{MN}\sum^{-1}K_{NM}\right)}^{-1}\;K_{MN}\sum^{-1}y$$

for N = M, Equation (7) simplifies to Equation (8):

(8) $$c\;=\;(K_{NN}\;+\;\sum {)}^{-1}y$$

**Figure 5:**
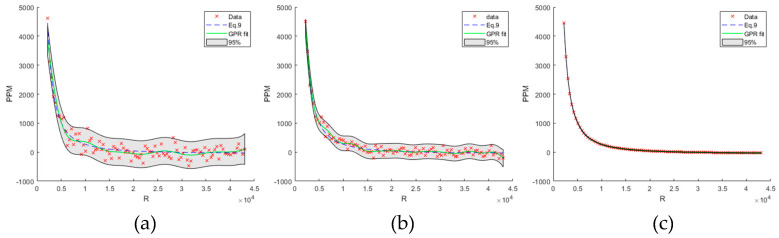
(**a**–**c**) GPR model with a squared exponential kernel trained on an exemplary ppm versus R curve (fixed T and RH), with added noise decreasing from left to right. The dotted blue line is the analytic model (Equation (9)). The GPR fit (green line) passes through the mean at each observational data point. The gray-shaded areas show the 95% confidence interval of the variance of the model predictions.

Equation (3) is the solution to Equation (8). As an example, Figure 5a–c show three GPR models with a squared exponential kernel trained on an exemplary ppm versus R curve, (fixed T and RH), with added noise decreasing from left to right (a–c). The dotted blue line is the analytic model (Equation (9)). The GPR fit (green line) passes through the mean at each observational data point. The gray-shaded areas show the 95% confidence interval of the variance of the model predictions.

(9) Cgas = (−1 + (RR0)−1β)/Kgas

### 2.3. Artificial Neural Nets (ANNs)

**Figure 6:**
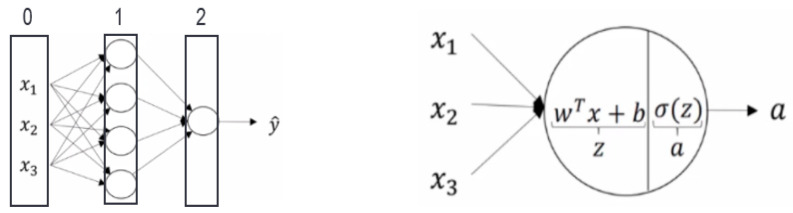
Illustration of shallow neural network with three inputs in the first layer (0) x_1_, x_2_ and x_3_, four nodes in the hidden layer (1), and one node in the output layer (2).

For background on ANNs, we refer the reader to the literature [17]. In brief, an exemplary neural network is shown in Figure 6 with three input features ***a***^0^***_j_*** = ***x_j_***, four nodes in a hidden layer, and one output ***a*** = $\hat{y}$ (Equation (10)). Regression minimizes the difference between $\hat{y}$ predicted and **y** measured with respect to the parameters ***w_jk_*** (weights) and ***b_j_*** (bias). A rectified linear unit (RELU) activation function was used throughout. Bayesian methods were used to automatically obtain the regularization parameters. Neural networks with one to three hidden layers and 10–100 nodes in each layer were trained using the same predictors that were used for the GPR models.

(10) $$z_{j}^{\left|l\right|}\;=\;\sum_{k\;=\;1}^{n^{\left|l\right|}}w_{jk}^{\left|l\right|}a_{k}^{\left|l\;-\;1\right|}\;+\;b_{j}^{\left|l\right|};\;a^{\left|l\right|}\;=\;g^{\left|l\right|}\left(z_{j}^{\left|l\right|}\right)$$

## 3. Results

### 3.1. Mean Absolute Errors

**Figure 7:**
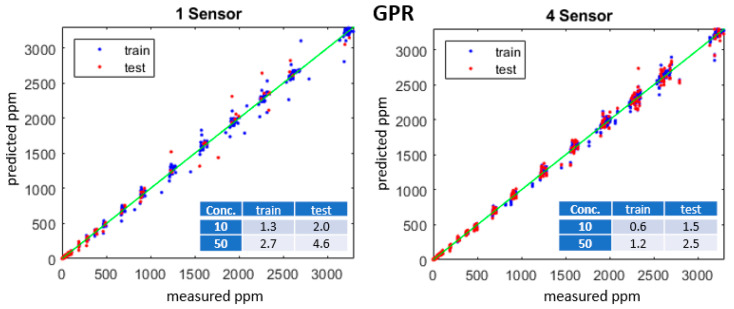
Predicted vs. measured methane concentrations (ppm) for training (blue) and test (red) datasets for one- and four-sensor GPR models with an isotropic exponential kernel. Table insets show the training and test MAE for 10 and 50 ppm.

**Figure 8:**
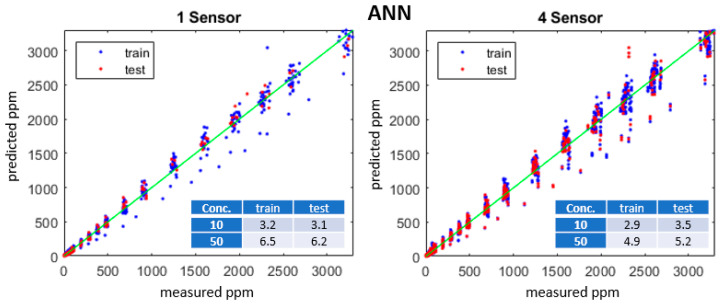
Predicted vs. measured methane concentration (ppm) for training (blue), test (red) and green line (one-to-one) datasets for one- and four-sensor ANN models with two inputs, two hidden layers, and 20 nodes, RELU activation. Table insets show the training and test MAE for 10 and 50 ppm.

Calibration datasets were split into training and test subsets using five-fold cross-validation to compute the average of the MAEs on the partitioned model. Individual MOx sensors exhibit variations in R; we trained models using up to 32 sensors. Figure 7 plots the predicted vs. measured ppm for GPR models with one or four sensors, an isotropic exponential kernel, and R, T, and RH predictors. Figure 8 plots the measured vs. predicted ppm for an ANN model with one hidden layer with 10 nodes. Insets in Figure 7 and Figure 8 list the MAE for each model at 10 and 50 ppm concentrations.

**Figure 9:**
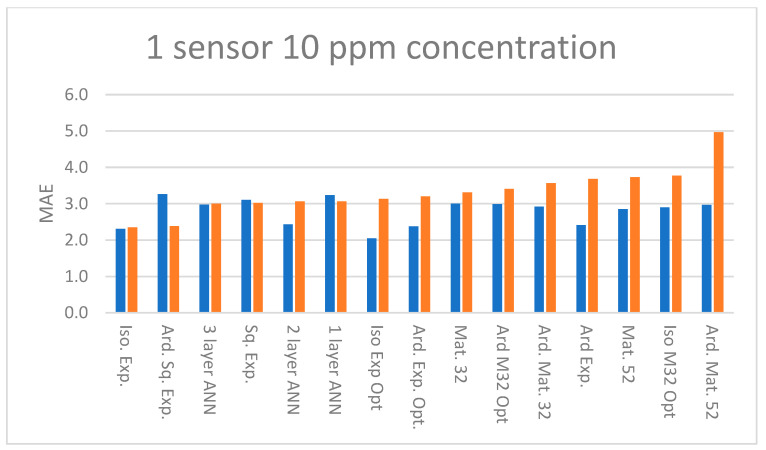
MAE of training (blue) and test (orange) sets at 10 ppm concentration with predictors R, T, and RH and response ppm for one sensor. See Table 1 for an explanation of abbreviations.

**Figure 10:**
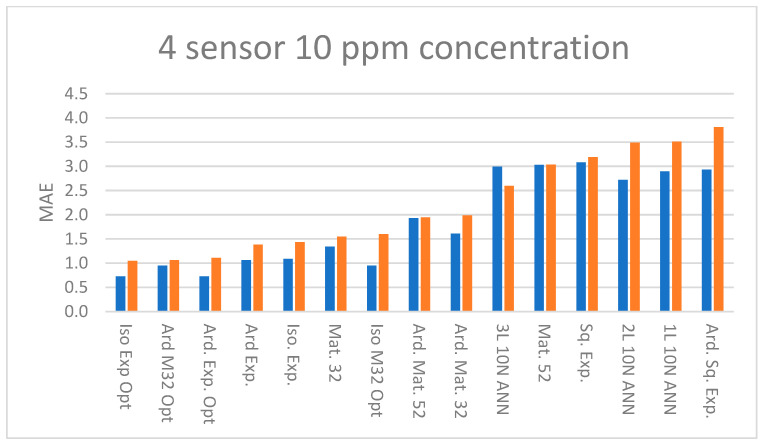
MAE of training (blue) and test (orange) sets at 10 ppm concentration with predictors R, T, and RH and response ppm for four sensors. See Table 1 for an explanation of abbreviations.

Figure 9 summarizes the MAE at 10 ppm concentration for one sensor GPR and ANN models. Figure 10 summarizes the MAE at 10 ppm concentration for four sensor GPR and ANN models. The abbreviations for the kernels and ANN are explained in Table 1. Models with the “opt.” suffix used Bayesian optimization in lieu of the LML method [16]. It is readily apparent that models built with four sensors achieve lower errors than models using only one sensor. 

### 3.2. Field Tests

**Figure 11:**
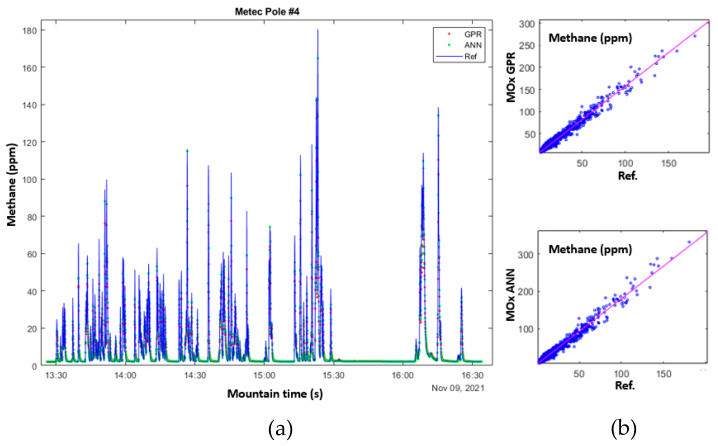
(**a**) Overlay of the response of a reference optical analyzer (blue line) with the MOx sensor response calibrated with GPR model (red dots) and ANN model (green dots) from a controlled leak test. (**b**) Correlation plots for reference analyzer and model calibrations for GPR (top), and ANN (bottom).

In field tests, the MOx sensors are mounted with temperature and humidity sensors in stainless steel housings with filters to prevent the accumulation of dust, water, and snow. The system has been deployed at two different outdoor facilities that can generate controlled releases of methane from a point. At least one sensor was installed alongside an optical reference sensor to enable comparing the response with ground truth and validate the calibration’s accuracy. One system was installed at the Colorado State University Methane Emissions Technology Evaluation Center (CSU METEC) facility. Figure 11a compares the response of an optical reference sensor (blue lines) with the MOx sensor calibrated using a GPR model (red dots) and ANN model (green dots). Figure 11b shows the correlation plots for each model vs. a reference sensor.

**Figure 12:**
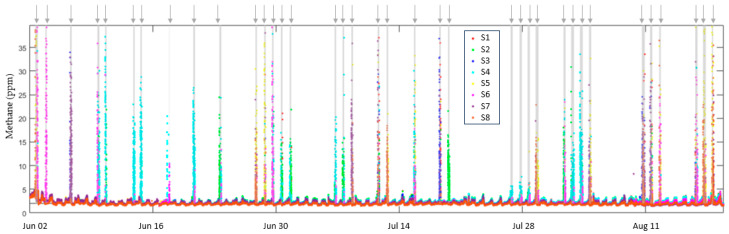
Response of eight sensors over a 3-month period. Grey-shaded regions show methane releases indicated by arrows. For each release, one or more sensors detected each release at the tens of ppm level, determined by the prevailing wind direction among other factors.

The second test installation was at the Oilfield Technology Center (OTC) operated by Texas Tech University in Lubbock. This facility is capable of large releases of up to 15 kg/h permitting us to validate the performance in the category of large emissions of more than 10 kg/h, a category known to produce at least 80% of total methane emissions [5]. Figure 12 shows the response of eight sensors over a 3-month period. Grey-shaded regions indicate specific periods when methane was released; each release is detected by at least one sensor.

**Figure 13:**
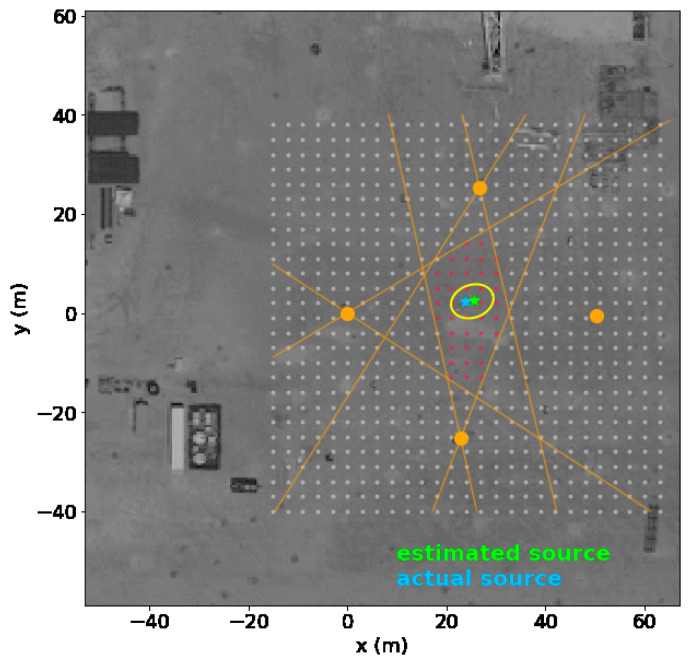
Interpreted results from 20 h of 9.3 kg/h releases showing the estimated source location (green) as compared to the actual location (blue) along with 95% confidence limits shown as the ellipse. The red region is the constrained source location area based on wind directions where methane concentrations above the background are detected by a given sensor as defined by the intersection of the orange constraints.

The data are streamed in real time to a cloud analysis platform where an inversion solver minimizes the mismatch between the observations and the predictive estimates from the forward model of the plume. The methane concentration is thereby linked to the emission source rate and location. Figure 13 shows the results for two different periods with approximately 20 h of releases each at this facility. The estimated leak position was calculated within 2.5 m of the source, with an inferred leak rate within 40% of the true value of 9.3 kg/h. An in-depth description of the solver is found in other publications [8,9].

### 3.3. Background Gases

**Figure 14:**
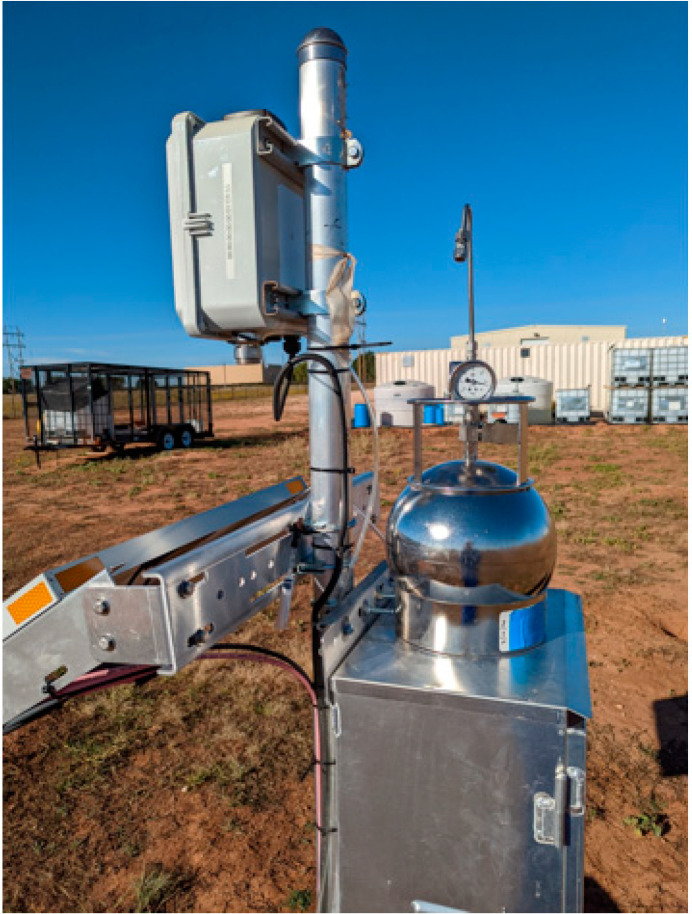
The 6L spherical metal canister, used for the background gas collection, is placed above the sensor unit to sample the background gases at the test site. Note that the background gas analysis was performed during our first generation of point instruments being used in the field, as depicted by the form factor of the unit in the picture.

The cloud inversion solver is effective at ruling out background from offsite sources. However, it is possible that hydrocarbon moieties present in natural gas (e.g., ethane), could lead to overestimation of the methane concentration and/or leak rate. To assess this risk, we tested background gases at the OTC in Lubbock, Texas. Passive air samples were collected over time in metal canisters mounted near the MOx sensors as shown in Figure 14. The canister was under vacuum prior to sampling, and through a flow-controlled regulator, it sampled at a constant rate for a 24 h period. A total of 70 compounds were measured using EPA Method TO-15 [18], Modified EPA Method 3C [19], and ammonia/siloxane sampling by other methods such as gas chromatography and mass spectrometry. The results for a subset of compounds are listed in Table 2; all are in the ppb to sub-ppb range. The reported values are below the levels at which they could cause significant dynamic interference with the methane measurements of MOx sensors. The results of the background gas analysis also suggest that typical concentrations of other compounds that could potentially be sensed by the MOx sensor are in much lower concentrations in the atmosphere and do not hinder the sensor’s performance in accurately detecting methane emissions in the field.

## 4. Discussion

**Figure 15:**
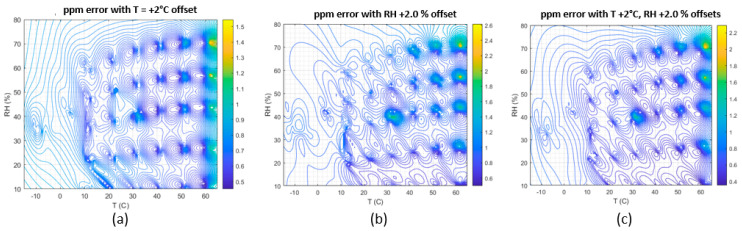
Sensitivity of the calibration model to offsets in the temperature (**a**) and relative humidity readings (**b**) and both (**c**). Apart from the edges of the calibration model, errors in the temperature +2 °C and RH +2.0% readings cause ppm errors of less than 1 ppm.

Modeling shows that a 1 kg per hour leak produces an average signal of 1 ppm at 50 m, depending on meteorological conditions [8,9]. Field tests detected leak rates below 1 kg/h. Therefore, it is essential that the system maintains a sensitivity of 1 ppm and that accuracy is not impacted by offsets in temperature or relative humidity. To assess the stability of the GPR models to offsets in temperature and relative humidity, we created a matrix of resistance (R) values on a grid of T and RH points spanning the environmental calibration range, assuming a background concentration of 2 ppm. A minimization procedure was used to calculate the difference between 2 ppm and the predicted ppm with a 2% T and/or RH offset applied. Figure 15a–c show that the error is on the order of 1 ppm, only slightly exceeding 1 ppm near the boundaries of the calibration region. A potential limitation is how well the calibration conditions translate into the dynamic environmental conditions seen in the field.

**Figure A1:**
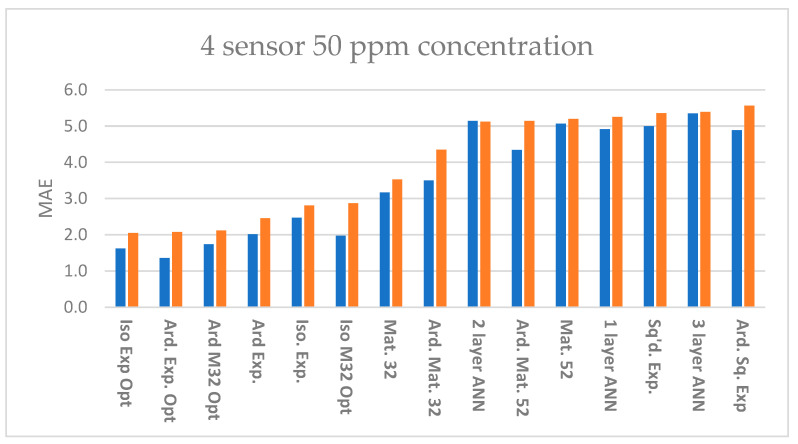
MAE of training (blue) and test (orange) sets at 50 ppm concentration with predictors R, AH, and response ppm for four sensors. Kernels for GPR models as labeled, the ANN models had 1–3 hidden layers each with 10 nodes per layer. See Table 1 for an explanation of abbreviations in the graph.

**Figure A2:**
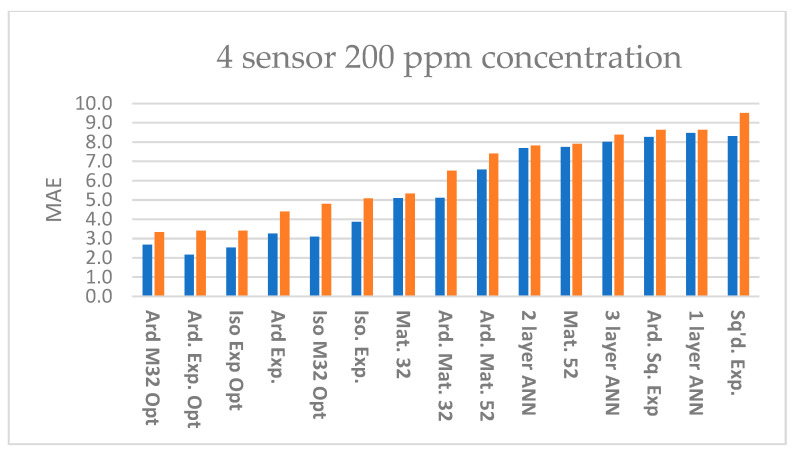
MAE of training (blue) and test (orange) sets at 200 ppm concentration with predictors R, AH, and response ppm for four sensors. Kernels for GPR models as labeled; ANN models labeled 1–3 hidden layers with 10 nodes per layer. See Table 1 for an explanation of abbreviations in the graph.

**Figure A3:**
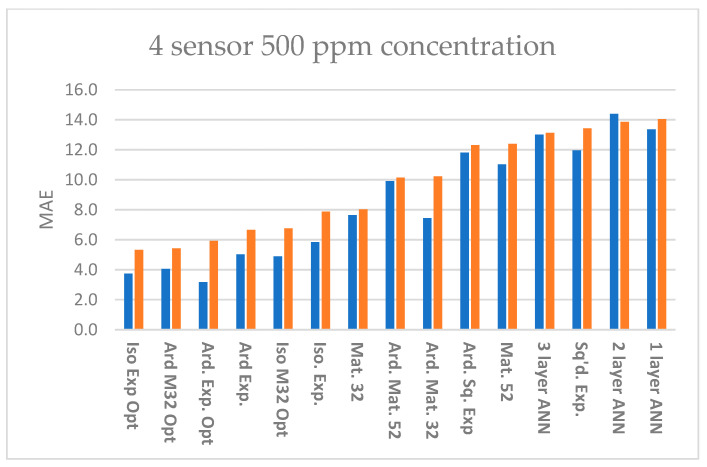
MAE of training (blue) and test (orange) sets at 500 ppm concentration with predictors R, AH, and response ppm for four sensors. Kernels for GPR models as labeled; ANN models labeled 1–3 hidden layers with 10 nodes per layer See Table 1 for an explanation of abbreviations in the graph.

**Figure A4:**
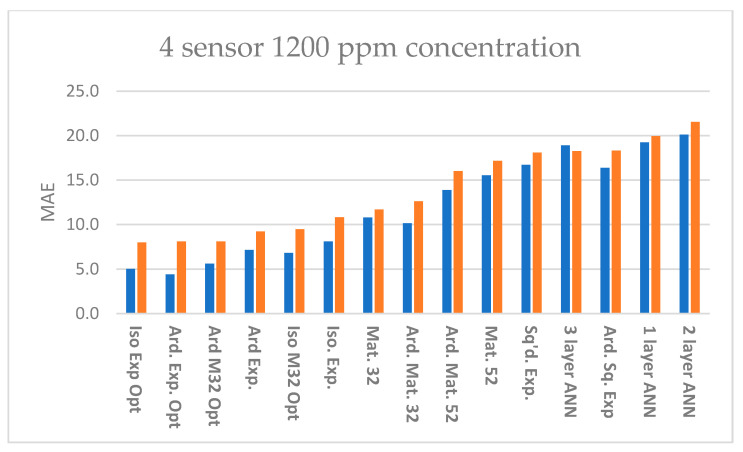
MAE of training (blue) and test (orange) sets at 1200 ppm concentration with predictors R, AH, and response ppm for four sensors. Kernels for GPR models as labeled; ANN models labeled 1–3 hidden layers with 10 nodes per layer. See Table 1 for an explanation of abbreviations in the graph.

**Figure A5:**
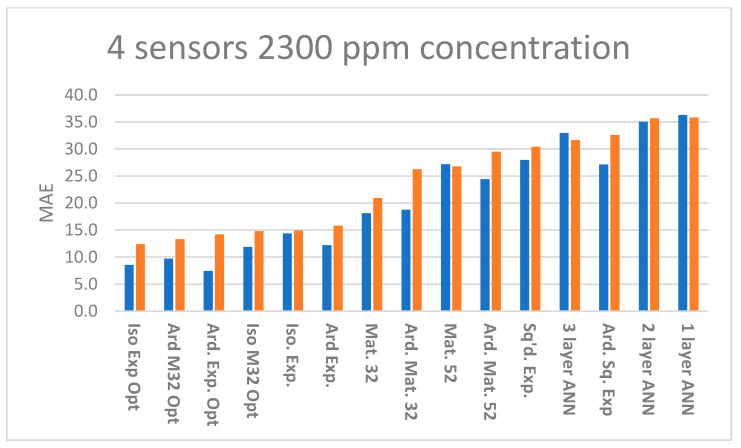
MAE of training (blue) and test (orange) sets at 2300 ppm concentration with predictors R, AH, and response ppm for four sensors. Kernels for GPR models as labeled; ANN models labeled 1–3 hidden layers with 10 nodes per layer. See Table 1 for an explanation of abbreviations in the graph.

Referring to Figure 9, one-sensor models achieve similar MAE. Referring to Figure 10, comparing four-sensor model’s MAE, the GPR models generally outperform the ANN models, and in Figure A1, Figure A2, Figure A3, Figure A4 and Figure A5 it is evident that this holds true at higher concentrations. The difference between the training and test set MAE is of the order of 1 ppm for the majority of four sensor models in the 10–200 ppm concentration range but at higher concentrations this increases to several ppm. Four-sensor Bayesian optimized models achieve lower MAE than models trained using the LML method, in both isotropic and ARD cases. However, comparing the training and test set MAE, the optimized and ARD GPR models appear to overfit the data at higher concentrations (Appendix A). A caveat is that the optimal kernel must be found by testing as it depends on the characteristics of the dataset.

## 5. Conclusions

For parametric models, incorporating the variability in sensor response between different batches without pre-screening large numbers of sensors can be challenging. ML models, on the other hand, can more easily incorporate an arbitrary number of sensors drawn from different batches. Moreover, ML models can be calibrated to accurately compensate for the dependence of MOx sensors on environmental conditions. Both goals are met with a minimum number of parameters, rendering the predictions more robust. The accuracy of GPR models compared with the ANN models increases with the concentration range. Multi-sensor GPR models perform better on average than ANN models with identical inputs and outputs. Therefore, GPR models are preferred over ANN models, especially at low concentrations (<10 ppm), where accurate leak rates are essential to pinpoint emissions. One advantage of GPR over ANN is that the kernel can be optimized with hyper-parameters, a trade-off between data fitting and smoothing not available with the latter. Small datasets, such as the MOx calibration set herein (*n* < 10,000), are computationally affordable.

In blind field tests, the point sensors were able to detect 97% of all the methane released and delivered a 0.6 kg/h limit of detection with a 90% probability of detection. Proposed governmental regulations require continuous monitors to detect emissions above a detection limit of approximately 1.0, so the performance of the point sensor is well aligned with the anticipated regulatory guidance. Combined with a cloud analysis platform, the use of ML for calibration of methane gas sensors improves both sensitivity and accuracy, with the potential to substantially reduce worldwide methane emissions.

## Figures and Tables

**Table 1:** Abbreviations used in Figure 9 and Figure 10, and Appendix A. For the ANN, LBFGS is the Broyden–Flecter–Goldfarb–Shanno quasi-Newton algorithm.

Abbreviation	Kernel Type	Hyperparameters	Optimization/Solver
Iso Exp Opt	Exponential	Isotropic	Bayesian
Ard M32 Opt	Matern 3/2	Nonisotropic	Bayesian
Ard. Exp. Opt	Exponential	Nonisotropic	Bayesian
Ard Exp.	Exponential	Nonisotropic	Log marginal Likelihood
Iso. Exp.	Exponential	Isotropic	Log marginal Likelihood
Mat. 32	Matern 3/2	Isotropic	Log marginal Likelihood
Iso M32 Opt	Matern 3/2	Isotropic	Bayesian
Ard. Mat. 52	Matern 5/2	Nonisotropic	Log marginal Likelihood
Ard. Mat. 32	Matern 3/2	Nonisotropic	Log marginal Likelihood
3L 10N ANN	3 layer, 10 nodes/layer	NA	LBFGS
Mat. 52	ARD Matern 5/2	Isotropic	Log marginal Likelihood
Sq. Exp.	Squared Exponential	Isotropic	Log marginal Likelihood
2L 10N ANN	2 layer, 10 nodes/layer	NA	LBFGS
1L 10N ANN	1 layer, 10 nodes/layer	NA	LBFGS
Ard. Sq. Exp.	Squared exponential	Nonisotropic	Log marginal Likelihood

**Table 2:** The table is a subset of the gases that were measured using the EPA TO-15 scan. The presence of these compounds at the Lubbock OTC site was negligible (~ppb range) and hence not of concern for cross-sensitivity for the MOx methane sensor.

Gases Detected:	6L Canister (ppb/V)
Propane	6.2
n-Butane	4.5
2-Methylbutane	3.2
Hexafluoropropene	2.9
Isobutane	2.9
Acetone	2.5
N-Pentane	1.7
2-Methylpentane	1
Hexamethylcyclotrisiloxane	0.6
Dichlorodifluoromethane (CFC 12)	0.44
2-Butanone	0.35
n-Hexane	0.35
Benzene	0.27
Trichlorofluoromethane (CFC 11)	0.19
Chloromethane	0.18
Methylene Chloride	0.075
Trichlorotrifluoroethane (CFC 113)	0.067
Ethylbenzene	0.029

## Data Availability

Data are contained within the article.

## References

[1] Hellgren L., Russell P., Fraioli S. (2023). Benchmarking Methane and Other GHG Emissions, Ceres. https://www.sustainability.com/contentassets/95c6e3e4c9a440049e3533575d0b389e/oilandgas_benchmarkingreport_2023.pdf.

[2] Beck C., Rashidbeigi S., Roelofsen O., Speelman E. (2020). The future is now: How oil and gas companies can decarbonize, McKinsey & Company. https://www.mckinsey.com/industries/oil-and-gas/our-insights/the-future-is-now-how-oil-and-gas-companies-can-decarbonize.

[3] Saunois M., Stavert A.R., Poulter B., Bousquet P., Canadell J.G., Jackson R.B., Raymon P.A., Dlugokencky E.J., Houweling S., Patra P.K. (2020). The global methane budget 2000–2017. Earth Syst. Sci. Data.

[4] Methane Tracker, IEA. 2023 Paris. https://www.iea.org/data-and-statistics/data-tools/methane-tracker.

[5] Brandt A.R., Heath G.A., Cooley D. (2016). Methane Leaks from Natural Gas Systems Follow Extreme Distributions. Environ. Sci. Technol..

[6] Cusworth D.H., Duren R.M., Thorpe A.K., Olson-Duvall W., Heckler J., Chapman J.W., Eastwood M.L., Helmlinger M.C., Green R.O., Asner G.P. (2021). Intermittency of Large Methane Emitters in the Permian Basin. Environ. Sci. Technol. Lett..

[7] Chakrabarti A., Dauphin M., Andrews A.B., Zielinski L., Rashid K., Yaun J., Speck A. Rapid Detection of Methane Super-Emitters through Advanced Interpretation. Proceedings of the SPE Annual Technical Conference and Exhibition.

[8] Andrews A.B., Boucher C., Chakrabarti A., Dauphin M., Doshi M., Rashid K., Speck A., van Pelt A., Yuan J., Zielinski L. Quantitative Mapping of Methane Emissions in Oil & Gas Facilities. Proceedings of the SPE Annual Technical Conference and Exhibition.

[9] Rashid K., Lukasz Zielinski L., Yuan J., Speck A. (2023). Subspace-Constrained Continuous Methane Leak Monitoring and Optimal Sensor Placement. arXiv.

[10] Shooshtari M., Vollebregt S., Vaseghi Y., Rajati M., Pahlavan S. (2023). The sensitivity enhancement of TiO2-based VOCs sensor decorated by gold at room Temperature. Nanotechnology.

[11] Potyrailo A., Go S., Sexton D., Li X., Alkadi N., Kolmakov A., Amm B., St-Pierre R., Schere B., Nayeri M. (2020). Extraordinary performance of semiconducting metal oxide gas sensors using dielectric excitation. Nat. Electron..

[12] Furuta D., Sayahi T., Li J., Wilson B., Presto A.A., Li J. (2022). Characterization of inexpensive metal oxide sensor performance for trace methane detection. Atmos. Meas. Tech..

[13] Abdullah A.N., Kamarudin K., Kamarudin L.M., Adom A.H., Mamduh S.M., Juffry Z.H.M., Bennetts V.H. (2022). Correction Model for Metal Oxide Sensor Drift Caused by Ambient Temperature and Humidity. Sensors.

[14] Thorson J., Collier-Oxandale A., Hannigan M. (2019). Using a low-cost sensor array and machine learning techniques to detect complex pollutant mixtures and identify likely sources. Sensors.

[15] Monroy J., Lilienthal A.J., Blanco J.L., González-Jiménez J., Trincavelli M. Calibration of MOX gas sensors in open sampling systems based on Gaussian Processes. Proceedings of the IEEE Sensors.

[16] Rasmussen C.E., Williams C.K.I. (2006). Gaussian Processes for Machine Learning.

[17] Bishop C.M. (1995). Neural Networks for Pattern Recognition.

[18] (1999). Organic, Determination of Volatile, and Specially-Prepared Canisters. Compendium of Methods for the Determination of Toxic Organic Compounds in Ambient Air.

[19] National Archives (2016). Revisions to Test Methods, Performance Specifications, and Testing Regulations for Air Emission Sources, Environmental Protection Agency. Fed. Regist. Dly. J. United States Gov..

